# Maternal exposure to nanoparticulate titanium dioxide during the prenatal period alters gene expression related to brain development in the mouse

**DOI:** 10.1186/1743-8977-6-20

**Published:** 2009-07-29

**Authors:** Midori Shimizu, Hitoshi Tainaka, Taro Oba, Keisuke Mizuo, Masakazu Umezawa, Ken Takeda

**Affiliations:** 1 Department of Hygienic Chemistry, Faculty of Pharmaceutical Sciences, Tokyo University of Science, Chiba 278-8510, Japan; 2 Research Center for Health Sciences of Nanoparticles, Research Institute for Science and Technology, Tokyo University of Science, Yamazaki 2641, Noda-shi, Chiba 278-8510, Japan

## Abstract

**Background:**

Nanotechnology is developing rapidly throughout the world and the production of novel man-made nanoparticles is increasing, it is therefore of concern that nanomaterials have the potential to affect human health. The purpose of this study was to investigate the effects of maternal exposure to nano-sized anatase titanium dioxide (TiO_2_) on gene expression in the brain during the developmental period using cDNA microarray analysis combined with Gene Ontology (GO) and Medical Subject Headings (MeSH) terms information.

**Results:**

Analysis of gene expression using GO terms indicated that expression levels of genes associated with apoptosis were altered in the brain of newborn pups, and those associated with brain development were altered in early age. The genes associated with response to oxidative stress were changed in the brains of 2 and 3 weeks old mice. Changes of the expression of genes associated with neurotransmitters and psychiatric diseases were found using MeSH terms.

**Conclusion:**

Maternal exposure of mice to TiO_2 _nanoparticles may affect the expression of genes related to the development and function of the central nervous system.

## Background

Nanotechnology and the production of novel man-made nanoparticles are increasing worldwide. Titanium dioxide (TiO_2_) has a high level of photocatalytic activity, and can be used for air and water purification and self-cleaning surfaces [1]. The activity level of nanoparticles is higher than that of bulk-sized particles [2,3]. TiO_2 _has the potential to produce reactive oxygen species (ROS) in its photocatalysis [1] and its possibly detrimental health effects are of concern. It has been reported that a mixture of anatase and rutile TiO_2 _nanoparticles induced cytotoxicity against human lung epithelial cells (BEAS-2B), even in the absence of photoactivation [4]. Sayes *et al*. [5] showed that anatase TiO_2 _nanoparticles, which can generate more ROS than rutile TiO_2 _particles, exhibited a higher level of cytotoxicity against human dermal fibroblasts and human lung epithelial cells (A549) than rutile TiO_2 _nanoparticles.

The small size of nanoparticles can bestow unique translocational properties [6,7]. It has been reported that nanosized elemental carbon particles (36 nm) inhaled by adult rats were translocated into extrapulmonary organs, such as liver [8]. A subsequent study showed that intranasally instilled carbon black nanoparticles can be translocated to the central nervous system, including cerebrum, cerebellum, and olfactory bulb via the olfactory nerve [9]. In a recent study, Takeda *et al*. [10] found that TiO_2 _nanoparticles administrated subcutaneously to pregnant mice were transferred from the mother to the fetal brain, and induced apoptosis in the mitral cells of the olfactory bulb of mice exposed maternally to the nanoparticles. Fetal brains are easily affected by blood-borne substances, including nano-sized materials, to a much greater extent than adult brains because the development of the blood-brain barrier in the fetal brains is incomplete [11]. Taking these observations into consideration, functional alterations of the central nervous system induced by maternal exposure to nanoparticles need to be investigated. To analyze the effect of maternal exposure to TiO_2 _nanoparticles on the early stages of development of the brain, we used microarray technology and gene expression profiles by functional annotation of genes using Gene Ontology (GO) terms and Medical Subject Headings (MeSH) terms.

## Methods

### Titanium dioxide nanoparticles

TiO_2 _nanopowder (particle size 25-70 nm; surface area 20-25 m^2^/g; crystal form anatase) was purchased from Sigma-Aldrich Japan Inc. (Tokyo, Japan) and used as TiO_2 _nanoparticles. The nanopowder was suspended in saline (Otsuka Pharmaceutical Factory Inc., Tokushima, Japan) with 0.05% (v/v) Tween 80 and sonicated for more than 30 minutes immediately before administration.

### Animals and treatments

Pregnant ICR mice, purchased from Japan SLC Inc. (Shizuoka, Japan), were housed in a room under controlled temperature (23 ± 1°C), humidity (55 ± 5%) and light (12 h light/12 h dark cycle with light on at 8:00 a.m.) with ad libitum access to food and water. Pregnant mice were transported carefully to minimize stress factors by Sankyo Labo Service Co., Inc (Tokyo, Japan). All animals were handled in accordance with institutional and national guidelines for the care and use of laboratory animals.

A 100 μL volume of TiO_2 _suspended at 1 μg/μL was injected subcutaneously into pregnant mice (*n *= 15) on gestational days 6, 9, 12, and 15 for the exposure group, while 100 μL of vehicle alone was injected into pregnant mice (*n *= 14) as a control group. Brain tissue was obtained from male fetuses on embryonic day (ED) 16 (*n *= 8/group) and from male pups on postnatal days 2 (*n *= 10/group), 7 (*n *= 10/group), 14 (*n *= 9/group), and 21 (*n *= 9/group).

### Total RNA extraction

Whole brains were immediately frozen in liquid nitrogen and kept at -80°C. Frozen tissue was homogenized and extracted with Isogen (Nippon Gene Co., Ltd., Tokyo, Japan) while well stirred by a Vortex-Genie 2 (Scientific Industries, Tokyo, Japan). Total RNA was isolated according to the manufacture's protocol and suspended in TE buffer (10 mM Tris-HCl, pH 8.0, 1 mM EDTA).

### Complementary DNA microarray analysis

RNAs for microarray analysis were pooled for each group, purified using the RNeasy Micro Kit (Qiagen, Hilden, Germany) and reverse-transcribed to yield complementary DNA (cDNA) labeled with the fluorescent dye Cy3 or Cy5 using the SuperScript Indirect cDNA Labeling Core Kit (Invitrogen, CA, USA) and the SuperScript Indirect cDNA Labeling System Purification Kit (Invitrogen). Cy3- and Cy5-labeled samples were purified using the CyScribe GFX Purification Kit (GE Healthcare Bio-Sciences, Little Chalfont, UK). The generated targets were mixed and subjected to hybridization to an NIA mouse 15 K Microarray v2.0 (AGC Techno Glass Co. Ltd., Chiba, Japan) consisting of 16,192 gene probes. Microarrays were scanned with two different photomultiplier sensitivities by a ScanArray (Packard BioChip Technologies, MA, USA). The scanner output images were normalized and signal quantification was performed using ScanArray Express (Perkin Elmer, MA, USA) and TIBCO Spotfire (TIBCO Software Inc., CA, USA). Normalization was used so that the overall intensity ratio of Cy3 and Cy5 was equal to 1. Statistical analysis was done with analysis of variance (ANOVA) and the level of statistical significance was set at *P *< 0.05.

### Functional analysis of microarray data with gene annotation

A total of 37 GO terms and 66 MeSH terms associated with anatomy, brain development and associated peptides, neurotransmitters, hormones, behavior and psychological phenomena, brain related disorders, oxidative stress, inflammation, and cell death were selected (Table 1, 2); and 2838 and 3625 genes were annotated by GO and MeSH terms, respectively, using the gene reference database PubGene (, Pub Gene AS, Oslo, NOR). These annotations were updated in April, 2008. The genes for which upregulation and downregulation were detected were categorized with GO and MeSH terms. The enrichment factor for each category was defined as (*nf*/*n*)/(*Nf*/*N*), where *nf *is the number of differentially expressed genes within the category, *n *is the total number of genes within that same category, *Nf *is the number of differentially expressed genes on the entire microarray, and *N *is the total number of genes on the microarray. Statistical analysis was performed using Fisher's exact test with hypergeometric distribution and the level of statistical significance was set at *P *< 0.05.

**Table 1:** List of GO terms selected for gene annotation

Category		GO term
biological process	developmental process	brain development
		forebrain development
		midbrain development
		hindbrain development
		generation of neurons
		glial cell differentiation
	
	biological regulation	cell death
		apoptosis
		neuron apoptosis
		activated T cell apoptosis
		B cell apoptosis
		negative regulation of neuron apoptosis
		apoptotic mitochondrial changes
		induction of programmed cell death
		induction of apoptosis
		anti-apoptosis
		glucocorticoid biosynthesis
		glucocorticoid metabolism
		neurotransmitter metabolism
		neurotransmitter transport
	
	multicellular organismal process	cognition
		learning and, or memory
	
	regulation of biological process	regulation of glial cell differentiation
		regulation of nerve growth factor receptor activity
		regulation of glucocorticoid biosynthesis process
	
	cellular process	mitochondrial fission
		mitochondrial fusion
	
	response to stimulus	response to oxidative stress
		response to reactive oxygen species
		response to superoxide
		superoxide metabolism
		glutathione biosynthesis
		glutathione metabolism

molecular function		motor activity
		superoxide dismutase activity
		glucocorticoids receptor activity
		brain derived neurotrophic factor binding

**Table 2:** List of MeSH terms selected for gene annotation

Category	MeSH term	
Anatomy	Blood Brain Barrier	Neurons
	Microglia	Olfactory Receptor Neurons
	Mitochondria	Synapses
	Neuroglia	

Diseases	Alzheimer Disease	Inflammation
	Anxiety Disorders	Learning Disorders
	Attention Deficit Disorder	Memory Disorders
	with Hyperactivity	Mitochondrial Disease
	Autistic Disorder	Neurogenic Inflammation
	Cognition Disorders	Parkinson Disease
	Epilepsy	Schizophrenia

Psychiatry and Psychology	Affective Symptoms	Memory
	Anxiety	Memory, Short-Term
	Cognition	Motivation
	Depression	Stress, Psychological
	Emotions	

Chemicals and Drugs	Apoptosis Inducing Factor	Anti-Anxiety Agents
	Apoptosis Regulatory Proteins	Glutathione
	Caspases	Glutathione Peroxidase
	Brain Derived Neurotrophic	Glutathione Synthase
	Factor	Inflammation Mediators
	Glial Cell Line-Derived	Neuronal Apoptosis-
	Neurotrophic Factor	Inhibitory Protein
	Nerve Growth Factor	Nitric Oxide
	Hormones	Reactive Oxygen Species
	Glucocorticoids	Superoxides
	Growth Hormone	Superoxide Dismutase
	Thyroid Hormones	

Neurotransmitters	Acetylcholine	Norepinephrine
	Dopamine	Serotonin
	Epinephrine	Receptors,
	gamma-Aminobutyric Acid	Neurotransmitter
	Glutamic Acid	Neuropeptides
		Neurotransmitter Uptake
		Inhibitors

Biological Science	Apoptosis	Motor Activity
	Cell Death	Neural Plasticity
	Gene, Mitochondrial	Oxidative Stress
	Lipid Peroxides	

## Results

### Analysis of cDNA microarrays

In the maternal TiO_2 _exposure group, the expression levels of 462 genes were changed significantly in the brain of the fetus at ED 16 (upregulation 229 genes; downregulation 233 genes), and those of 864 (upregulation 234; downregulation 630), 417 (upregulation 351; downregulation 66), 738 (upregulation 450; downregulation 288), and 1887 (upregulation 613; downregulation 1274) were changed significantly in the brain of offspring 2, 7, 14, and 21 days old, respectively (Table 3). The number of genes differentially expressed between groups was increased remarkably in the brain of 21 days old pups.

**Table 3:** The number of genes differentially expressed in maternal TiO_2 _exposure group

Age	Upregulated	Downregulated	Total
Embryonic day 16	229	233	462

2 days old	234	630	864

7 days old	351	66	417

14 days old	450	288	738

21 days old	613	1274	1887

### Functional categorization of microarray data

Of the genes expressed differentially in the maternal TiO_2 _exposure group, 3, 2, 8, and 4 GO categories were enriched significantly in the brain at 2, 7, 14, and 21 days after birth, respectively (Table 4), while 6, 2, 36, and 28 MeSH categories were enriched significantly at 2, 7, 14, and 21 days after birth (Additional file 1). Eight MeSH categories were also enriched significantly in the fetal brain at ED 16 (Additional file 1). The largest group of GO categories enriched was those related to cell death 2 - 21 days after birth; 121 and 64 genes linked to apoptosis at 2 and 7 days after birth, respectively, and 92 and 173 genes linked to "cell death" were identified at 14 and 21 days after birth. "Brain development" was also a large category at 2 (34 genes) and 14 (43 genes) days after birth. GO categories related to oxidative stress, such as "superoxide dismutase activity", were also enriched significantly at 14 and 21 days after birth. The largest MeSH categories enriched were "Mitochondria" at ED 16 (31 genes) and 2 days (56 genes) after birth and "Apoptosis" at 14 (118 genes) and 21 (230 genes) days after birth. The "Mitochondria" category was persistently enriched at 14 (60 genes) and 21 (109 genes) days after birth. MeSH categories related to oxidative stress, such as "Glutathione", "Lipid Peroxidation", and "Reactive Oxygen Species", were also enriched significantly at ED 16 and 14 and 21 days after birth. MeSH categories related to inflammation and neurotransmitters including "Epinephrine", "Norepinephrine", "Serotonin", and "Glutamic Acid" were also highly enriched at 14 and 21 days after birth.

**Table 4:** Significantly enriched GO categories in maternal exposure group vs. control group

GO term	Enrichment factor	P value
**Embryonic day 16**		
(None)		
**2 days old**		
apoptosis	1.04	.05
brain development	1.21	.04
motor activity	1.80	.02
**7 days old**		
apoptosis	1.11	.01
glial cell differentiation	5.14	.02
**14 days old**		
activated T cell apoptosis	3.75	.02
brain development	1.48	.00
cell death	1.08	.04
induction of apoptosis	1.28	.01
motor activity	1.58	.05
response to oxidative stress	1.70	.01
response to reactive oxygen species	1.53	.05
superoxide dismutase activity	2.22	.01
**21 days old**		
anti-apoptosis	1.58	.02
cell death	1.03	.04
glutathione biosynthesis	1.62	.04
superoxide dismutase activity	1.75	.01

## Discussion

Nanoparticles have a high level of reactivity with biological tissue, since they have a large specific surface area [6,7]. It has been reported that fullerenes, which are manufactured carbon nanoparticles, induce oxidative stress in the brain of juvenile largemouth bass [12]. Tin-Tin-Win-Shwe *et al*. [13] showed that intranasal instillation of ultrafine carbon black (14 nm) to mice induced a higher level of expression of cytokines and chemokines in the olfactory bulb compared to those induced by the same mass of carbon black (95 nm). The particles used in the exposed pregnant mice group can enter the circulatory system and can transfer to and damage the fetus. Sugamata *et al*. [14] reported that the cytoplasmic granules of granular perithelial cells contain particles of diesel exhaust (DE) and degenerate in both the cerebral cortex and the hippocampus of mice exposed prenatally to DE. A later study [15] showed that maternal DE exposure alters the levels of monoamines and their metabolites in brains and spontaneous motor activity in male mice. Since TiO_2 _was detected in the brain of mice maternally exposed to TiO_2 _nanoparticles [10], which is the material used in this study, microarray was applied to the analysis of the effects of maternal exposure to TiO_2 _nanoparticles on the brain of neonatal mice.

In the present study, we used only male fetuses and pups for analysis because the prevalence of some psychiatric disorders in childhood, such as autism and attention deficit hyperactivity disorder, is higher in men than in women. The results of the microarray analysis showed changes in expression of hundreds of genes in the brain at ED 16, and at 2, 7, 14, and 21 days after birth. To interpret the large amount of data generated, functional categorization using GO terms and MeSH terms were performed, which identified potentially important categories on the basis of both a high enrichment factor (>1.00) and statistical significance (*P *< 0.05). MeSH is a controlled vocabulary thesaurus produced by the National Library of Medicine and used for indexing, cataloging, and searching for biomedical and health-related information and documents. Although most researchers use GO for providing annotation to genes, MeSH terms are proposed to be a useful complementary tool for interpretation of microarray data [16]. A subsequent report [17] showed that the use of MeSH has the advantage of producing anatomical and disease information with respect to the genes of interest. In the present study, genes were annotated with the terms related to anatomy, brain development, brain-related disorders, those associated with nanotoxicology (oxidative stress [6,7,12] and inflammation [6,7,13]), and those associated with the effects of maternal exposure to DE or TiO_2 _nanoparticles (hormones [18], behavior and neurotransmitters [15,18], and cell death [10,14,19]) for analysis.

As a result, GO terms associated with development of brain were extracted at 2 and 14 days after birth, those associated with cell death, including apoptosis, were extracted 2 to 21 days after birth, and those associated with response to oxidative stress were extracted at 14 and 21 days. Brain development is regulated by neurotrophins such as nerve growth factor, brain-derived neurotrophic factor [20], and glial cell line-derived neurotrophic factor [21], and hormones including growth hormone [22] and thyroid hormone [23,24]. Analysis using MeSH terms showed that alteration of these factors that can lead to abnormal development of the central nervous system was induced by maternal exposure to TiO_2 _nanoparticle. It has been reported that neuronal cell death, including apoptosis, is essential for elimination of neurons and axons to make correct synaptogenesis in the early stage of brain development [25,26]. The result of functional analysis suggested that disruption of these processes can be caused by maternal exposure to TiO_2 _nanoparticles.

It has been reported that the changes of environment surrounding pregnant mice cause abnormal level of neurotransmitters in the brain of the offspring. Meyer *et al*. [27] reported that maternal immune challenge by the viral mimic polyriboinosinic-polyribocytidilic acid causes abnormal fetal dopaminergic development, which is similar to a schizophrenic symptom. Maternal stress also induces altered expression of genes related to the dopaminergic system in the midbrain and causes hyperactivity in adult offspring [28]. The results that MeSH terms associated with neurotransmitters and motor activity were extracted suggest that maternal exposure to TiO_2 _nanoparticles causes abnormal levels of neurotransmitters that can lead to altered motor activity.

As for MeSH terms, those associated with diseases were extracted in the functional analysis. Some diseases such as autistic disorder, epilepsy, and learning disorders, occur in childhood, and although Alzheimer's disease, schizophrenia, and Parkinson's disease arise mainly in adulthood or old age, related MeSH terms were extracted in the results from infant mice of mothers exposed to TiO_2_. In the early 1990s, Dr David Barker J.P. stated that fetal undernutrition increases the incidence of cardiovascular disease in adult life [29]. Subsequent studies showed the environment that the fetus senses indirectly through the mother can be linked to other diseases in adulthood, and proposed a hypothesis of "early developmental origins of adult disease" [30]. The results of the present study suggest that maternal exposure to nanoparticles can alter gene expression in the neonatal period and might cause the onset of psychiatric disorders even in adulthood. However, the present study did not show how the maternal response to the nanoparticles altered the mother's behavior toward the pups and how this in turn altered gene expression. Further investigations are needed to clarify the critical factor for the gene expression change. Moreover, the changes caused by maternal exposure to TiO_2 _nanoparticles should not be limited to the brain. Our published [10] and unpublished data suggest that other organ systems are also affected.

## Conclusion

**Figure 1:**
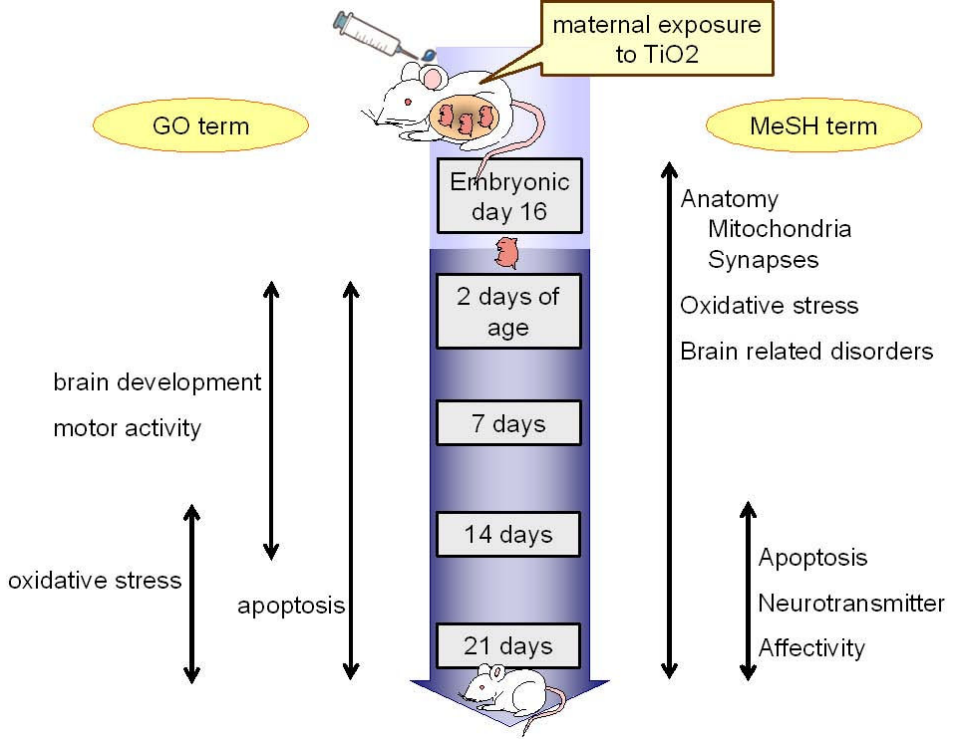
**Summary of the extracted terms with genes differentially expressed in the maternal TiO_2 _exposure group**.

This study showed that maternal exposure to anatase TiO_2 _nanoparticle caused the changes in the expression of genes associated with brain development, cell death, response to oxidative stress, and mitochondria in the brain during the perinatal period, and those associated with inflammation and neurotransmitters in the later stage (Figure 1). Further investigation is needed to clarify the alterations of neurotransmitter levels and motor function. This study showed also that analysis using microarray data with GO and MeSH terms can provide meaningful information, and will contribute to further interpretation of microarray results in toxicological research.

## Abbreviations

cDNA: complementally DNA; DE: diesel exhaust; ED: embryonic day; GO: Gene Ontology; MeSH: Medical Subject Headings; ROS: reactive oxygen species; TiO_2_: titanium dioxide.

## Competing interests

The authors declare that they have no competing interests.

## Authors' contributions

KT conceived the overall research idea. MS, TO, and KM carried out all procedure for animal experiments. HT, an expert on microarray analysis, had idea to apply GO and MeSH term methods for study of gene expression. MS and HT conducted the microarray analysis. MU participated substantially in the functional analysis of microarray data and drafted the manuscript. All authors read and approved the final manuscript.

## Supplementary Material

Additional file 1 **Significantly enriched MeSH categories in maternal exposure group vs. control group**. Additional table. Click here for file
