# Supplementary material for: Maternal exposure to nanoparticulate titanium dioxide during the prenatal period alters gene expression related to brain development in the mouse
Source: Part Fibre Toxicol. 2009 Jul 29;6:20. doi: 10.1186/1743-8977-6-20 (PMC2726979; doi:10.1186/1743-8977-6-20)
Supplement: Additional file 1 — Significantly enriched MeSH categories in maternal exposure group vs. control group. Additional table. [file 1743-8977-6-20-S1.doc]

**Additional file 1. Significantly enriched MeSH** categories in maternal exposure group vs. control group

| MeSH term | Enrichment factor | P value |
| --- | --- | --- |
| Embryonic day 16  Alzheimer Disease  Glutathione Peroxidase  Glutathione  Lipid Peroxidation  Microglia  Mitochondria  Nitric Oxide  Reactive Oxygen Species  2 days old  Memory Disorders  Mitochondria  Neuropeptides  Schizophrenia  Stress, Psychological  Synapses  7 days old  Anti-Anxiety Agents  Schizophrenia  14 days old  Alzheimer Disease  Apoptosis Inducing Factor  Apoptosis  Autistic Disorder  Brain Derived Neurotrophic Factor  Caspases  Cell Death  Epilepsy  Epinephrine  Glial Cell Line-Derived Neurotrophic Factor  Glucocorticoids  Glutamic Acid  Glutathione Synthase  Growth Hormone  Hormones  Inflammation Mediators  Inflammation  Learning Disorders  Lipid Peroxidation  Memory  Mitochondria  Mitochondrial Diseases  Motor Activity  Nerve Growth Factor  Norepinephrine  Olfactory Receptor Neurons  Oxidative Stress  Parkinson Disease  Reactive Oxygen Species  Schizophrenia  Serotonin  Stress, Psychological  Superoxide Dismutase  Superoxides  Synapses  Thyroid Hormones  21 days old  Apoptosis Regulatory Proteins  Apoptosis  Caspases  Cell Death  Cognition  Dopamine  Epilepsy  Epinephrine  gamma-Aminobutyric Acid  Glutamic Acid  Glutathione Peroxidase  Glutathione  Inflammation  Learning Disorders  Lipid Peroxidation  Mitochondria  Neuroglia  Neurons  Neuropeptides  Nitric Oxide  Norepinephrine  Reactive Oxygen Species  Receptors, Neurotransmitter  Serotonin  Stress, Psychological  Superoxide Dismutase  Superoxides  Synapses | 1.49  1.67  1.30  1.66  1.49  1.18  1.52  1.39  1.75  1.12  1.34  2.12  1.54  1.30  3.60  2.25  1.66  2.02  1.07  2.44  1.58  1.24  1.31  2.38  1.56  1.59  1.29  1.46  2.84  1.47  1.69  1.54  1.27  2.56  1.51  1.29  1.20  1.98  1.59  2.03  1.64  2.12  1.17  1.90  1.40  2.99  1.99  1.69  1.58  1.75  1.31  1.61  1.27  1.03  1.13  1.08  1.38  1.37  1.40  1.34  1.40  1.23  1.34  1.16  1.14  1.50  1.18  1.08  1.10  1.002  1.10  1.11  1.26  1.19  1.44  1.18  1.47  1.28  1.33  1.18 | .03  .03  .04  .02  .03  .05  .00  .02  .04  .04  .01  .01  .05  .03  .04  .05  .00  .03  .03  .04  .01  .01  .00  .00  .01  .05  .03  .01  .02  .01  .00  .01  .01  .00  .01  .05  .02  .05  .00  .00  .01  .00  .03  .00  .00  .00  .00  .03  .00  .00  .03  .03  .01  .03  .02  .03  .04  .00  .02  .01  .01  .01  .01  .02  .01  .04  .04  .03  .04  .04  .04  .03  .03  .01  .03  .04  .02  .00  .01  .03 |
